# Phytochemical Analysis and Biological Activities of Flavonoids and Anthraquinones from *Cassia alata* (Linnaeus) Roxburgh and Their Implications for Atopic Dermatitis Management

**DOI:** 10.3390/plants14030362

**Published:** 2025-01-24

**Authors:** Sue-Kei Lee, Jing-Wen Keng, Jessica-Ai-Lyn Yon, Chun-Wai Mai, Heng-Chee Lim, Sek-Chuen Chow, Gabriel Akyirem Akowuah, Kai Bin Liew, Siew-Keah Lee, Philip J. Marriott, Yik-Ling Chew

**Affiliations:** 1 Faculty of Pharmaceutical Sciences, UCSI University, Kuala Lumpur 56000, Malaysia; 1001644199@ucsiuniversity.edu.my (S.-K.L.); 1001851267@ucsiuniversity.edu.my (J.-W.K.); 1001953303@ucsiuniversity.edu.my (J.-A.-L.Y.); 1002161890@ucsiuniversity.edu.my (H.-C.L.); 2 Centre for Cancer and Stem Cell Research, Institute for Research, Development and Innovation (IRDI), IMU University, Kuala Lumpur 57000, Malaysia; chunwai_mai@imu.edu.my; 3 School of Science, Monash University Malaysia, Jalan Lagoon Selatan, Bandar Sunway, Subang Jaya 46150, Selangor, Malaysia; chow.sek.chuen@monash.edu; 4 School of Pharmacy, Monash University Malaysia, Jalan Lagoon Selatan, Bandar Sunway, Subang Jaya 46150, Selangor, Malaysia; gabriel.akowuah@monash.edu; 5 Faculty of Pharmacy, University of Cyberjaya, Persiaran Bestari, Cyber 11, Cyberjaya 63000, Selangor, Malaysia; liewkaibin@cyberjaya.edu.my; 6 M. Kandiah Faculty of Medicine and Health Sciences, Universiti Tunku Abdul Rahman, Jalan Sungai Long, Bandar Sungai Long, Kajang 43000, Selangor, Malaysia; leesiewkeah@utar.edu.my; 7 Australian Centre for Research on Separation Science, School of Chemistry, Monash University, Wellington Road, Clayton, VIC 3800, Australia

**Keywords:** *Cassia alata*, polyphenols, wound healing, antioxidant, antimicrobial, atopic dermatitis

## Abstract

To study *Cassia alata* (CA) (Linnaeus) Roxburgh’s effectiveness towards atopic dermatitis (AD), CA leaf extracts were prepared using three methanol-based extraction solvent systems. Bioactive constituents were characterized and quantified via high-performance liquid chromatography with diode array detection. Antioxidant properties and antimicrobial activities against *Staphylococcus aureus*, a major AD exacerbation factor, were assessed. Four polyphenols (two flavonoids, two anthraquinones) beneficial in AD control were detected (rhein > aloe-emodin > astragalin > kaempferol). The 75% *v*/*v* MeOH/water extract had the most polyphenols and the best antioxidant profile (total phenolic content, total flavonoid content, 2,2-diphenyl-1-picrylhydrazyl-hydrate radical scavenging activity, ascorbic acid equivalent antioxidant capacity), with excellent *S. aureus* inhibition (minimum inhibitory concentration = 0.625 mg/mL; minimum bactericidal concentration = 1.25 mg/mL). Hence, it was selected for the in vitro examination of cytotoxicity and wound healing activity towards human epidermal keratinocyte cells using a 3-(4,5-dimethyl-2-thiazolyl)-2,5-diphenyl-2h-tetrazolium bromide (MTT) assay and wound scratch assay. The extract showed no cytotoxicity (IC_50_ > 100 µg/mL) without significant reduction in cell viability up to 200 µg/mL compared to the vehicle control. An amount of 50 μg/mL extract concentration showed the best wound-healing activity (*p* < 0.05), with a cell migration rate of 5.89 ± 0.80 µm/h over 96 h post-treatment. Such antioxidant, antimicrobial, and wound-healing activities suggest CA and its polyphenols to be promising natural, long-term AD remedies for skin health.

## 1. Introduction

Eczema or atopic dermatitis (AD) is a prevalent chronic skin disorder among children and some adults, commonly characterized by itching, dry skin, and lichenification [1]. AD patients often suffer from an increased risk of skin and systemic infections, immune dysregulation, and *Staphylococcus aureus* skin colonization [2]. Excessive scratching during exacerbation can injure the skin, causing delayed wound closure and re-epithelialization [3].

The link between oxidative stress and AD is well documented. Simonetti et al. [4] found increased lipid peroxidation and serum lipoprotein peroxidation along with lower myeloperoxidase, paraoxonase 1, and paraoxonase 2/3 levels in AD patients, all of which are oxidative stress indicators. Formulations with known antioxidative plant extracts [5] have shown promising effects in AD management and the alleviation of oxidative stress, which is important in the treatment of AD.

Current first-line therapies, including topical corticosteroids and non-steroidal immune-modulating agents, have been found to have side effects, including burning, itching, and skin infection [6]. Thus, patients with severe eczema might be more likely to use complementary and alternative medicine [7]. One common herb used for treating the condition is *Cassia alata* (Linnaeus) Roxburgh.

*C. alata* (L.) Roxb. (CA) or *Senna alata* (L.) Roxb. is a tropical shrub commonly known as candle bush, *daun gelenggang*, or *daun kurap* in Malay; *ath thora* or *Eth Thora* in Sri Lanka; and *dad mardan* in India [8]. The plant has been used traditionally to treat skin diseases. It is a traditional Chinese treatment for eczema, itching, neurodermatitis, and skin abscesses, where the macerated leaves are applied topically to the skin [9]. This effect may be attributed to its antibacterial, antifungal, anti-inflammatory, and antioxidant properties [10].

Scientific evidence regarding the mechanism of CA in AD management is lacking. This paper addresses this by examining the phytochemical content and evaluating the antibacterial effect on *S. aureus*, antioxidant, and wound-healing properties of CA leaf extracts. The leaves were extracted with the methanol (MeOH) of different concentrations and characterized using HPLC with diode array detection (HPLC–DAD), then assessed for antioxidant activity and antimicrobial effects on *S. aureus*. These tests were used to select the best extract for the in vitro scratch assay. HPLC–DAD allows for rapid, versatile compound detection via correlation with standard reference compounds in the chromatogram and spectra at varying wavelengths [11], and thus it was used in the phytochemical analysis of the extract.

## 2. Results

### 2.1. HPLC–DAD Identification and Quantification of Important Bioactive Constituents in CA Extracts

**Figure 1:**
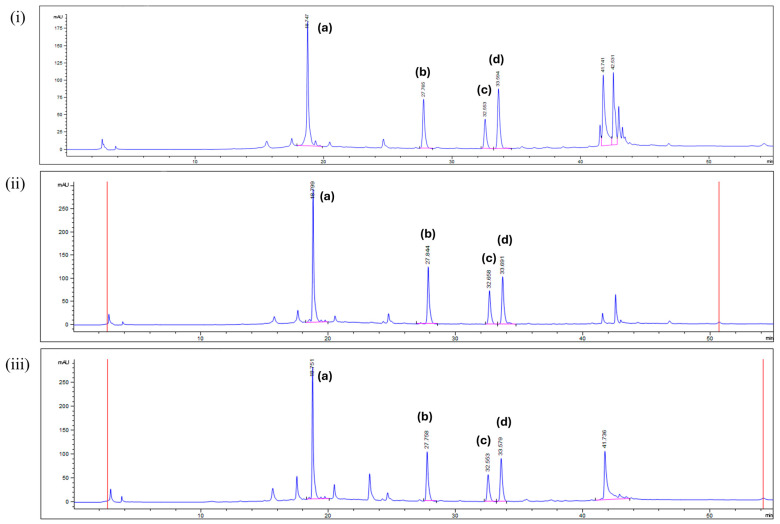
HPLC chromatograms of CA extracts prepared using (**i**) 100% *v*/*v* MeOH, (**ii**) 75% *v*/*v* MeOH, and (**iii**) 50% *v*/*v* MeOH at 254 nm. Peaks of important bioactive constituents are labeled as follows: (a) astragalin; (b) kaempferol; (c) aloe-emodin; (d) rhein.

Four important polyphenols potentially useful for managing AD were detected using HPLC–DAD in all CA extracts: astragalin, kaempferol, aloe-emodin, and rhein, eluted at retention times (*t*_R_) of 18.8 min, 27.8 min, 32.6 min, and 33.6 min, respectively. Figure 1 reports the relative proportions of various compounds in the different MeOH extracts.

**Figure 2:**
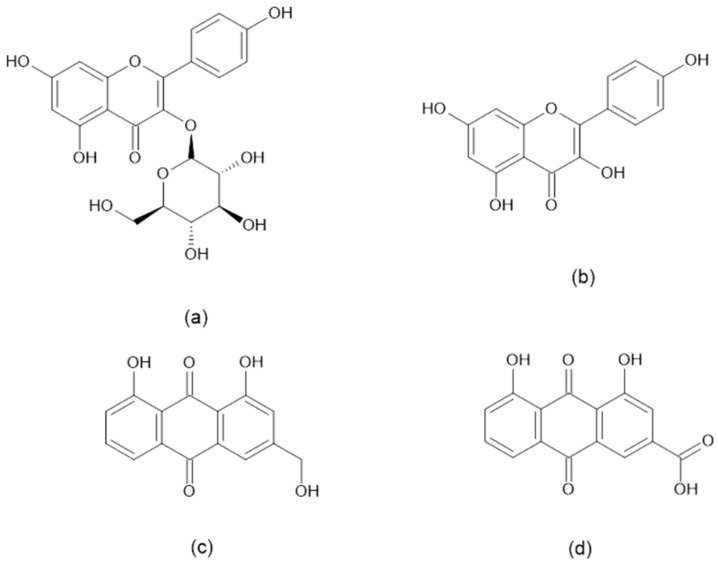
Chemical structures of important bioactive constituents detected in CA extracts: (**a**) astragalin; (**b**) kaempferol; (**c**) aloe-emodin; (**d**) rhein.

**Figure 3:**
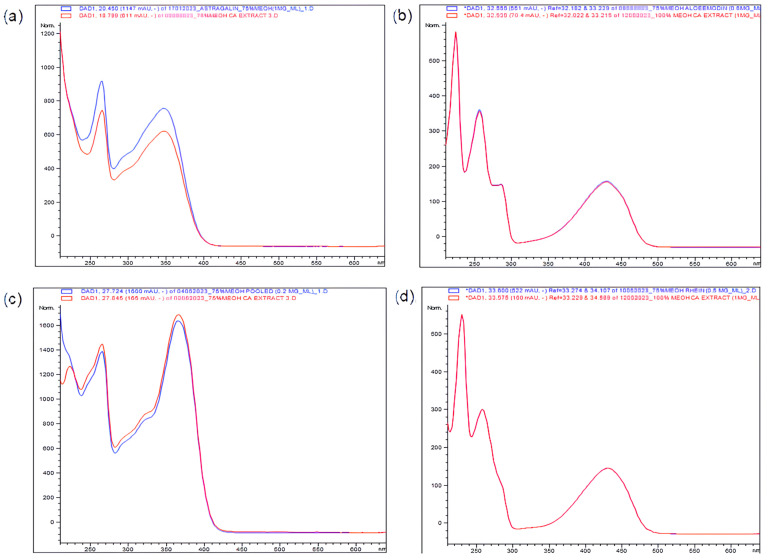
UV spectra overlay between (**a**) astragalin; (**b**) kaempferol; (**c**) aloe-emodin; (**d**) rhein in extract samples, and their respective standard reference peaks. UV spectra in blue are for samples; UV spectra in red are for standards.

Kaempferol and astragalin are flavonoids. Meanwhile, aloe-emodin and rhein are anthraquinones with high structural resemblance (Figure 2) [12]. High amounts of these flavonoids and anthraquinones were also previously reported in CA leaves [12,13,14,15]. The UV spectra of astragalin, kaempferol, aloe-emodin, and rhein were compared against their respective standards (Figure 3).

CA leaves were used historically to treat AD worldwide [10]. The folkloric use of CA in AD management could be based on the presence of these four important bioactive constituents. These polyphenols may act synergistically to give rise to significant antioxidant, antimicrobial, wound healing, and anti-inflammatory effects, which could be beneficial for AD management [13,16,17,18,19].

To compare the concentration and composition of astragalin, kaempferol, aloe-emodin, and rhein contained in 1 mg/mL of the extracts, quantification was carried out using HPLC–DAD at 254 nm as shown in Table 1. Standard calibration curves for these constituents were constructed as follows: astragalin (y = 35,240x, R^2^ = 0.9982); kaempferol (y = 52,023x, R^2^ = 0.9902); aloe-emodin (y = 7018.4x, R^2^ = 0.9977); rhein (y = 6876.9x, R^2^ = 0.9991).

All CA extracts contained these four constituents in the order of rhein > aloe-emodin > astragalin > kaempferol. In all CA extracts, rhein was present in the greatest amount. This finding was in accordance with previously published studies [12,14,15]. Interestingly, the rhein content in this current study was much higher than in the previous studies (between 0.02 and 0.15% *w/w*) [14]. This could be due to environmental factors, including the geographical regions, climates, and growth conditions of CA, which could affect the accumulation of rhein in the leaves [20]. Post-harvest handling and drying and extraction solvents used for CA leaf extraction could also contribute to rhein content variation in the extracts [21,22].

HPLC analysis of CA extracts in this current study also showed that 75% *v*/*v* MeOH exhibited better recoveries (*p* < 0.05) of all four polyphenols of interest from CA leaves, followed by 50% *v*/*v* MeOH and, lastly, 100% *v*/*v* MeOH. Similar findings were demonstrated in Barchan et al.’s study, which reported that the combination of MeOH and water was found to have extracted a relatively higher quantity of polyphenols from the leaves of three *Mentha* species relative to other solvent systems [23].

### 2.2. In Vitro Antioxidant Evaluation of CA Extracts

Oxidative stress characterized by reactive oxygen species (ROS) overproduction by epidermal keratinocytes could cause skin barrier damage and trigger inflammation leading to the development of AD [9,24,25]. CA leaves rich in polyphenols could be a good source of natural antioxidants to supplement the skin’s antioxidant defense system.

As shown in Table 2, all CA extracts showed high total phenolic content (TPC) and total flavonoid content (TFC). Generally, 75% *v*/*v* MeOH CA extract showed significantly higher (*p* < 0.05) TPC and comparable (*p* > 0.05) TFC compared to 100% *v*/*v* MeOH CA extract. Both TPC and TFC were significantly (*p* < 0.05) lower in 50% *v*/*v* MeOH CA extract. Such results did not correspond well with the concentration of polyphenols recorded in Table 1. This suggested that TPC and TFC measures may include other polyphenols that were not characterized in this current study. Another explanation would be that the Folin–Ciocalteu phenol reagent, which primarily measures the reducing capacity of a sample in the TPC assay, may also be reduced by reducing species other than phenolic compounds [26]. Owing to this lack of specificity for phenolic compounds, the TPC assay result may not reflect the actual TPC of a plant extract [26,27]. The TFC assay using aluminum chloride that works based on chelation between Al(III) metal ions and flavonoids—supposedly at a 1:1 ratio—also has some limitations. Not all flavonoids will form complexes with Al(III) and may not have the same absorption maxima (λ_max_) at the region of interest. Hence, the TFC of a plant extract may not be depicted accurately [28]. Therefore, the results obtained from these assays were complemented by and compared with the results from other in vitro antioxidant assays to correlate with their characteristic antioxidant activities.

Free radicals, such as reactive oxygen species (ROS) generated during the pathogenesis of AD, are believed to contribute to lipid peroxidation, antioxidant depletion, and oxidative damage to the skin cell membrane. [9]. To determine the free radical scavenging properties of CA extracts, a 2,2-diphenyl-1-picrylhydrazyl-hydrate (DPPH) radical scavenging assay was conducted and expressed as the inhibitory concentration of DPPH radicals at 50% (IC_50_) and ascorbic acid equivalent antioxidant capacity (AEAC). According to Table 2, significantly strong DPPH radical scavenging activity (*p* < 0.05) was exhibited in 75% *v*/*v* MeOH CA extract, while comparable activity (*p* > 0.05) was noted for both 100% *v*/*v* MeOH and 50% *v*/*v* MeOH CA extracts. Similar patterns of results were noticed for TPC, IC_50_, and AEAC: 75% *v*/*v* MeOH > 100% *v*/*v* MeOH > 50% *v*/*v* MeOH CA extract. There was a strong negative association between TPC and IC_50_ (R^2^ = 0.9980)_._ A strong positive association at the same magnitude was observed between TPC and AEAC (R^2^ = 0.9980). Similar correlations of these antioxidant properties were also noticed in tropical seaweeds and herbs from the Leguminosae family [29,30]. This indicates that most of the phenolic compounds in CA extracts can act as a free radical scavenger, which means that they donate protons to free radicals (DPPH•, in this context). Rhein and aloe-emodin in the extracts are prominent antioxidants that showed significant concentration-dependent free radical scavenging activity, as reported by Vargas et al. [31]. Park et al. reported that astragalin and kaempferol, with their free radical scavenging actions, were shown to protect cellular membranes from lipid peroxidation better than (+)-α-tocopherol [32]. Moreover, the free radical scavenging activity of rhein and kaempferol was also found to be more potent than antioxidant vitamins such as vitamins C and E commonly used in skin care products [31,32]. Hence, the rich content of antioxidants in these extracts will make CA leaves a promising natural remedy to relieve oxidative stress and overcome skin inflammation associated with AD.

CA extracts also showed promising ferric-reducing antioxidant power (FRAP). Significant differences in FRAP (*p* < 0.05) were noticed in the extracts. Interestingly, the FRAP in this study did not follow a similar pattern of results as their respective TPC despite a very strong positive correlation between TPC and FRAP (R^2^ = 0.9999). There was a slight but significant discrepancy (*p* < 0.05) between TPC and the corresponding FRAP values for 100% *v*/*v* MeOH and 75% *v*/*v* MeOH CA extracts. Like the TPC assay, the FRAP assay works based on the reducing ability of a sample through single-electron transfer. However, the FRAP assay is not specific to polyphenolic antioxidants. Hence, a significantly higher FRAP value in 100% *v*/*v* MeOH CA extract could be attributed to other non-antioxidant reductants present in the extract, which led to a false positive result in this assay [33].

In AD patients, a high serum level of malondialdehyde (MDA) was significantly associated with the severity of AD [9,34]. MDA is a product of lipid peroxidation or an oxidant that could upset the prooxidant–antioxidant balance [9]. Hence, supplementation with antioxidants from CA extracts could potentially alleviate AD by restoring the prooxidant–antioxidant balance. The results in Table 2 suggested that, in addition to the flavonoids and anthraquinones characterized in this current study, other antioxidative compounds present in the CA extracts could potentially exhibit good reducing power to delay the formation of MDA and thus ameliorate skin condition in AD.

**Figure 4:**
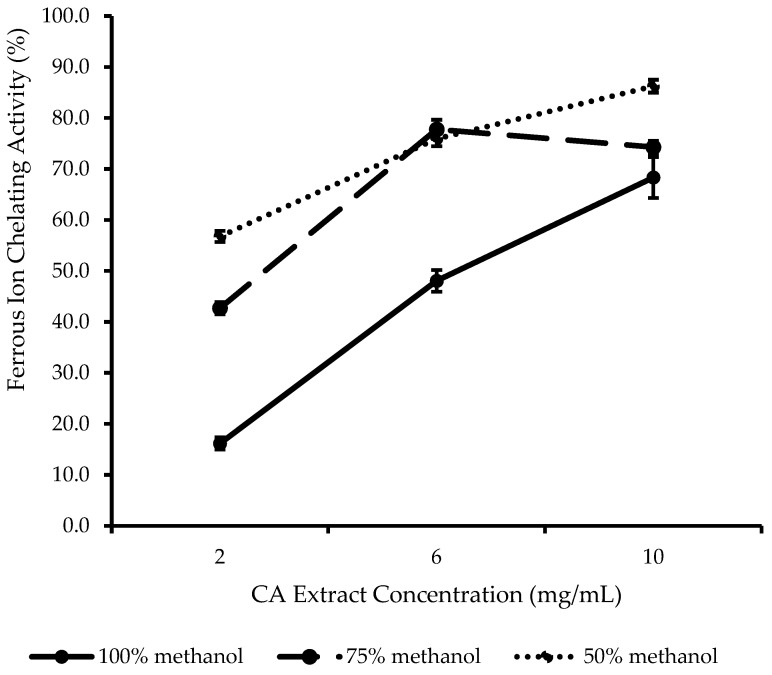
Ferrous ion chelating (FIC) activity of CA extracts prepared using 100% *v*/*v* MeOH, 75% *v*/*v* MeOH, and 50% *v*/*v* MeOH. Results are expressed as mean ± SD (*n* = 3).

The metal-chelating ability of antioxidants in the extracts was assessed by using a ferrous ion chelating (FIC) assay (Figure 4). Unlike all the other assays, 50% *v*/*v* MeOH CA extract showed the highest FIC activity, followed by 75% *v*/*v* and, lastly, 100% *v*/*v* MeOH CA extract. Neither TPC nor TFC showed an apparent association with FIC activity. This finding was in agreement with the observation reported by Loh and Lim [35], who proposed that not all antioxidative phenolic compounds extracted from avocado leaves have the ability to form chelates with the ferrous ion.

There was a significant difference in FIC activity (*p* < 0.05) between 100% *v*/*v* and 50% *v*/*v* MeOH at all CA extract concentrations. FIC activities were comparable (*p* > 0.05) between 75% *v*/*v* MeOH and 50% *v*/*v* MeOH at 6 mg/mL, and between 100% *v*/*v* MeOH and 75% *v*/*v* MeOH at 10 mg/mL. The FIC activities of CA extracts could be mainly due to the presence of flavonoids and anthraquinones characterized in this current study. Rhein, the most significant anthraquinone detected in CA extracts in this current study, is a bidentate metal chelator known to chelate metal ions via its carbonyl and hydroxyl groups [36,37]. Aloe-emodin contains hydroxymethyl groups at position C3 and hydroxyl groups at position C1 and C8. Aloe-emodin was reported by Yen [38] as a chain-breaking antioxidant with substantial metal-chelating activity. Flavonoids such as astragalin and kaempferol contain multiple sites for metal chelation and could also reduce redox-active metal-catalyzed ROS production, thus potentially protecting the skin from oxidative stress and further skin inflammation associated with AD [39,40]. Hence, it could be deduced that the findings from Figure 4 were consistent with the results in Table 1, which showed that the extract prepared using 100% *v*/*v* MeOH had the lowest overall content of the four important constituents detected. Meanwhile, rhein in 75% *v*/*v* MeOH and 50% *v*/*v* MeOH CA extracts only differed slightly, which justifies the slight difference in FIC activity between these two extracts. Another explanation may be that the slight but significantly higher FIC activity of 50% *v*/*v* MeOH CA extract at 2 mg/mL and 10 mg/mL could also be attributed to other metal-chelating antioxidants present but not characterized in this current study.

Based on the overall findings, 75% *v*/*v* MeOH CA extract demonstrated strong and favorable antioxidant properties in terms of TPC, TFC, and DPPH radical scavenging activity, as well as FRAP and FIC activity when compared to the others. The excellent antioxidant profile of CA leaves is attributable to the four significant constituents detected, along with other antioxidative compounds present in the extract. The flavonoids and anthraquinones characterized in this current study possessed great versatility in their antioxidant mechanism, which includes free radical scavenging, metal chelating, and reducing properties (ability to donate H-atoms and electrons). Such antioxidant properties would be advantageous in AD management, as supplementation with these antioxidants could help to strengthen the antioxidant defense system and subsequently relieve oxidative stress and skin inflammation.

### 2.3. Evaluation of Antimicrobial Efficacy of CA Extracts Against S. aureus ATCC 25923

Massive colonization of *S. aureus* is a significant factor contributing to secondary skin infection, which could result in AD flares and exacerbation [9]. It could intensify skin inflammation in AD by releasing δ-toxins and pro-inflammatory lipoproteins that adversely damage keratinocytes and stimulate cytokine release and immune cell expansion in the skin [41]. When compared to healthy individuals, *S. aureus* could easily adhere to and colonize the skin of AD patients [41]. Therefore, controlling *S. aureus* colonization on the skin with an antimicrobial agent would be a feasible approach to address AD-related inflammation, especially during flares.

In this current study, all CA extracts exhibited excellent antimicrobial potency with a minimum bactericidal concentration (MBC)/minimum inhibitory concentration (MIC) ratio of ≤4 against *S. aureus* ATCC 25923 strain (Table 3). This result was in agreement with previous studies conducted using the ethanolic and aqueous extracts of CA leaves [42,43,44].

In a previous report by Doughari and Okafor [45], a methanolic extract of CA leaf was found to inhibit *S. aureus* at MIC and MBC of 12 mg/mL. The significant difference in MIC and MBC compared with this current study could be due to the content variation in antimicrobial constituents present in CA leaves, as they were separately sampled from different geographical regions at different times [46].

The pronounced antibacterial activity of CA extracts against *S. aureus* was thought to be primarily due to the presence of anthraquinones and flavonoids in the extracts [47]. Aloe-emodin and kaempferol recovered from CA leaves demonstrated excellent antibacterial activity against multidrug-resistant *S. aureus*, with MIC = 12.0 μg/mL and 13.0 μg/mL, respectively [17]. Aloe-emodin could retard the formation of *S. aureus* bacterial biofilms by inhibiting extracellular protein production at impressively low MIC and MBC of 4 µg/mL [48,49]. Kaempferol could inhibit sortase A, a bacterial transpeptidase, which would anchor to specific surface proteins of the *S. aureus* cell wall. It weakens the binding and adhesion of *S. aureus* to fibrinogen, thus inhibiting biofilm formation [50]. Rhein was also reported to possess a bacteriostatic effect against *S. aureus* with MIC = 12.5 µg/mL, which was able to impair the virulence of *S. aureus* by disrupting the biofilm matrix and also biofilm formation, besides inhibiting hemolysin and catalase activities [51]. Astragalin was also known to inhibit *S. aureus* growth, although its antimicrobial potency was reportedly lower with MIC = 83.0 µg/mL [17].

According to Table 3, *S. aureus* was about two times more sensitive to 75% *v*/*v* MeOH CA extract than to 100% and 50% *v*/*v* MeOH CA extracts, as evidenced by the lowest MIC (0.625 mg/mL) and MBC (1.25 mg/mL) values. This result was consistent with the TPC shown in Table 2. However, this did not correspond well with the content of four bioactive constituents, as shown in Table 1. This suggested that the antimicrobial activity of the extracts was not solely dependent on the four bioactive constituents (astragalin, kaempferol, aloe-emodin, and rhein) but could also be a result of synergism among other antibacterial polyphenols present in the extract but not characterized in this current study.

CA leaves have been a natural antibacterial remedy for various bacterial skin infections. Despite having a lower potency than the conventional antibiotic ampicillin, a great antimicrobial potency (MBC/MIC = 2) against *S. aureus* was evident in 75% *v*/*v* MeOH CA extract. This current finding suggests that CA leaves, with their rich source of antimicrobial constituents, could be a safer and more effective natural alternative to conventional antibiotics for addressing *S. aureus* infection [52]. Through adequate suppression of *S. aureus* skin colonization, AD flares and exacerbation could potentially be reduced, thus justifying the usefulness of CA leaves in AD management.

### 2.4. Selection of Best Solvent for C. alata Leaf Extraction

Organic solvents are frequently used to extract plant materials due to the high extraction efficiency of bioactive polyphenols [35]. Previous studies suggested that MeOH and ethanol (EtOH) were the preferred organic solvents for extracting hydrophilic polyphenols like those present in CA leaves [14,53,54,55]. MeOH showed a superior extraction yield, with high specificity and effectiveness in extracting polyphenols of lower molecular weights compared to EtOH [29]. Hence, MeOH was selected as the extraction solvent for CA leaves in this current study. The extraction yield of CA leaves extracted using 100%, 75%, and 50% *v*/*v* MeOH were comparable, which were 22.5%, 19.4%, and 22.5%, respectively.

Previous studies involving the methanolic extraction of CA leaves were inconclusive regarding the optimum concentration of MeOH used [53,56]. Considering the overall results obtained from the HPLC–DAD analysis and antioxidant and antimicrobial studies in this current study, 75% *v*/*v* MeOH was selected as the best extraction solvent relative to 100%, and 50% *v*/*v* MeOH and was used for further in vitro cell-based assays.

### 2.5. Evaluation of Cytotoxicity and Wound Healing Efficacy of 75% v/v MeOH CA Extract in the Immortalized Human Keratinocytes (HaCaT) Cell Line

AD is often associated with serious itching, whereby vigorous scratching could deleteriously disrupt skin anatomical continuity and functionality, resulting in an open wound [57,58]. However, uncontrolled skin inflammation in AD could contribute to extensive wound formation and delayed wound healing, making wound care more challenging for AD patients [3,59]. Most current evidence regarding the wound-healing properties of CA leaf extracts was limited to in vivo models only [57,60]. Only in vitro wound healing effects of CA leaf extracts were reported by Agampodi and Collet [53] using a morphological assay and a direct cell proliferation assay in immortalized human keratinocytes (HaCaT) cell line. In contrast, the wound healing efficacy of 75% *v*/*v* MeOH CA extract was studied using an in vitro scratch wound healing assay in this study, which involves the deliberate creation of a scratch on confluent HaCaT cells before treatment for a better illustration of the wound-healing effect of the extract [53].

**Figure 5:**
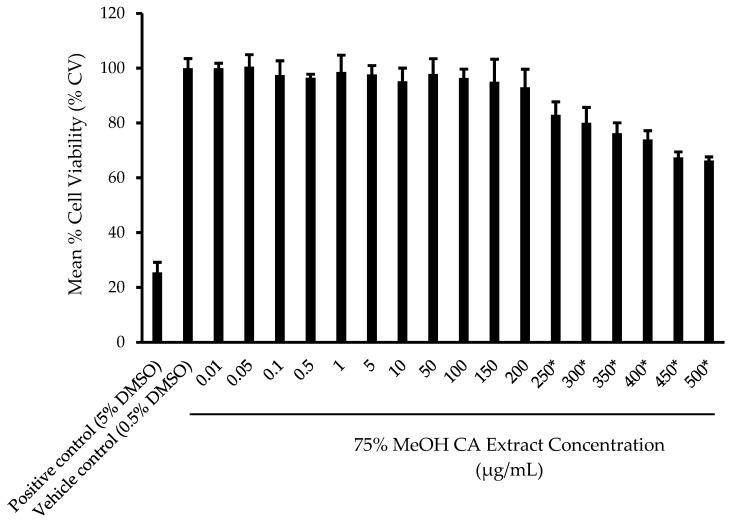
Mean percentage (%) cell viability of HaCaT cells upon treatment using 75% *v*/*v* MeOH CA extract at different concentrations. Results are expressed as mean ± SD (*n* = 3). Statistical differences (*p* < 0.05) as measured by paired samples *t* test between vehicle control and treated samples are indicated by the * (asterisk) symbol. Note: DMSO, dimethyl sulfoxide.

To eliminate the possibility of the reduction of cell viability due to the direct toxicity of the CA extract, a 3-(4,5-dimethyl-2-thiazolyl)-2,5-diphenyl-2h-tetrazolium bromide (MTT) assay was first carried out by using a range of extract concentrations (0.01–500 µg/mL). From Figure 5, no significant reduction in HaCaT cell viability (*p* > 0.05) was observed at concentrations up to 200 µg/mL of the extract compared to the vehicle control. The predicted concentration of the extract, which resulted in a 50% reduction in cell viability (IC_50_) value, was 769 µg/mL, in agreement with that of Saidin et al. [61], which showed that both the ethanolic and aqueous extracts of CA leaves exhibited IC_50_ values ranging from 145.43 µg/mL to >1000 µg/mL in WRL-68 (human liver) and Vero (African green monkey kidney) cell lines, thus confirming their lack of cytotoxic effects. Amounts of 10, 50, 100, and 200 µg/mL were selected as the treatment concentrations of the extract for the subsequent scratch assay.

**Figure 6:**
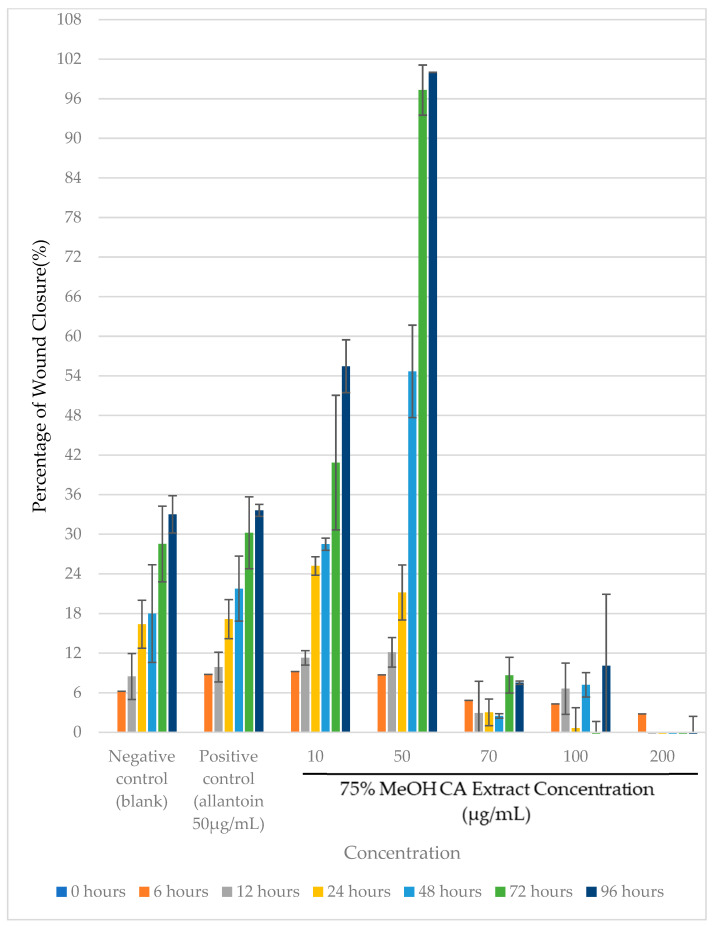
Mean percentage (%) wound closure with time of HaCaT cells upon treatment using different concentrations of 75% *v*/*v* MeOH CA extract.

According to the results from Figure 6, two out of five tested concentrations of the extract showed a significant percentage of wound closure (*p* < 0.05) compared to the negative control. Notably, it exhibited the best wound closure (*p* < 0.05) at 50 µg/mL, with complete (100%) wound closure at 96 h post-treatment. A significant difference (*p* < 0.05) in the percentage of wound closure was noticeable between 75% *v*/*v* MeOH CA extract and allantoin at the same treatment concentration (Figure 6). This indicates that the extract may have higher wound-healing potency than the conventional wound care product, allantoin at the same concentration.

**Figure 7:**
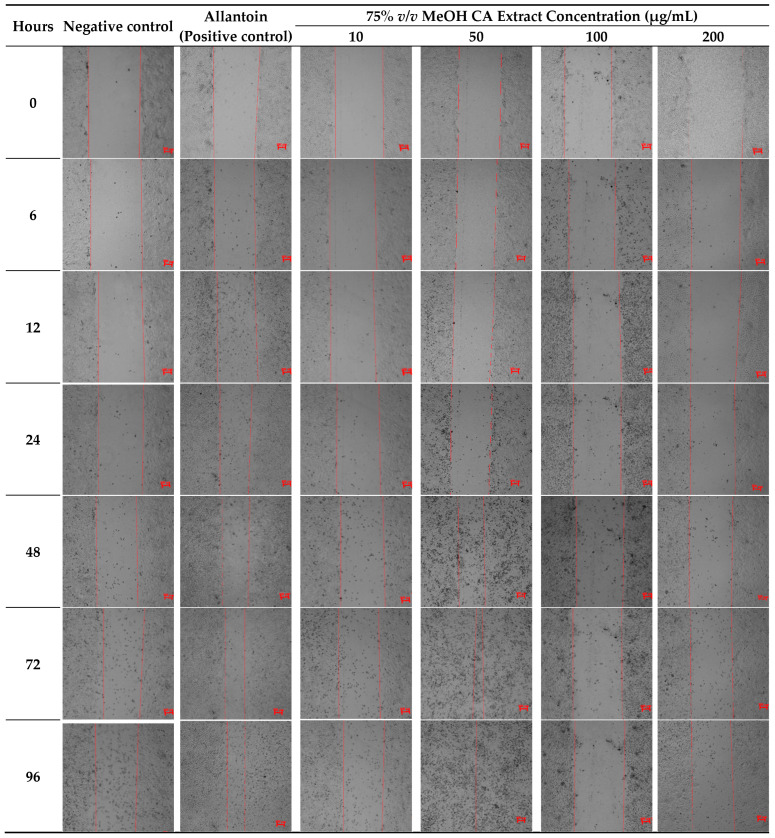
Microscopy images of wound closure of HaCaT cells using different concentrations of 75% *v*/*v* MeOH CA extract taken at 0, 6, 12, 24, 48, 72, and 96 h post-treatment. Allantoin (50 µg/mL) was used a positive control.

Similarly, based on observations from Figure 7, the wound scratch treated with 50 µg/mL of the extract became invisible as early as 48 h post-treatment compared to both the negative and positive controls. Also, the cell density of HaCaT cells treated with 50 µg/mL of the extract appeared higher compared to the negative control. The wound closure increased exponentially. This suggested that 75% *v*/*v* MeOH CA extract at 50 µg/mL could accelerate the wound-healing process, possibly by increasing the proliferation rate of HaCaT cells. This finding was in agreement with Agampodi and Collet [53].

**Figure 8:**
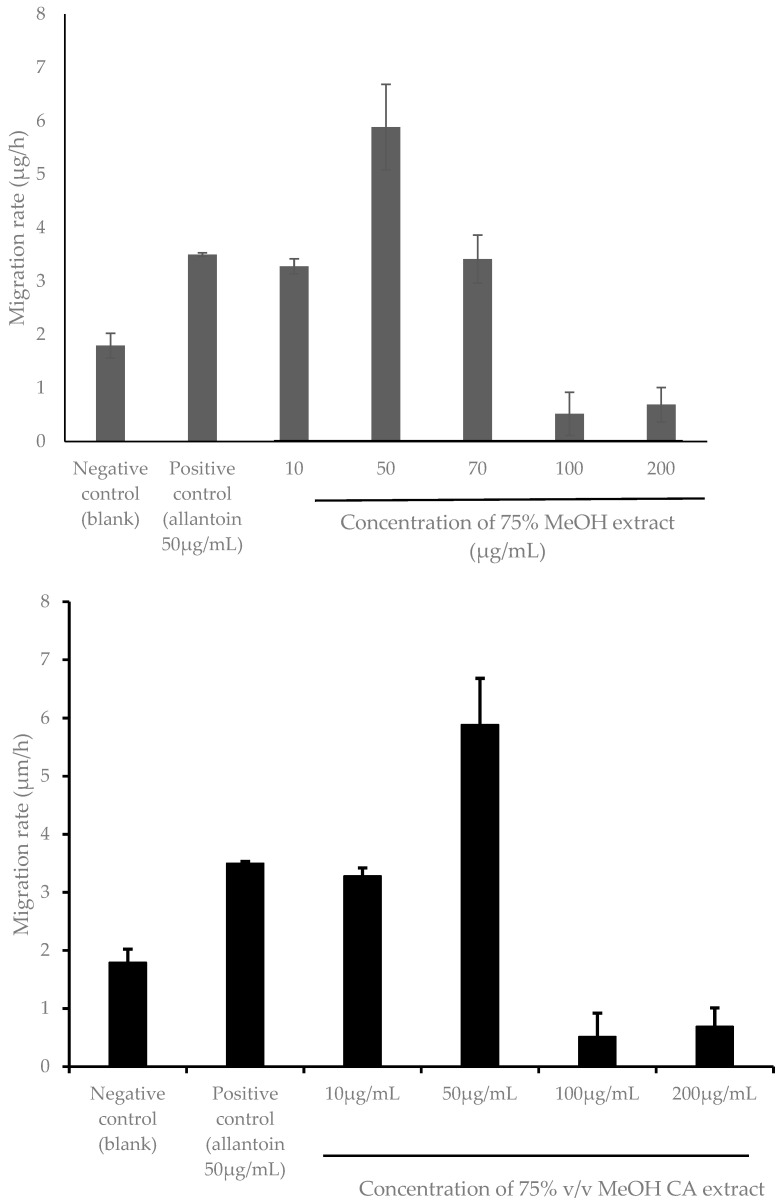
Mean migration rate of HaCaT cells at 96 h post-treatment using different concentrations of 75% *v*/*v* MeOH CA extract. Results are expressed as mean ± SD (*n* = 3).

Wound healing is a complex process that consists of several phases: hemostasis, inflammation, proliferation, and tissue remodeling [57]. During proliferative phase of wound healing, lateral cell migration takes place where keratinocytes move as a layer across the wound to form a complete and intact monolayer. Keratinocyte cell migration, which occurs during this phase, is a rate-limiting step in skin re-epithelialization [62,63]. Consistent with the results in Figure 6, the 75% *v*/*v* MeOH CA extract exhibited the highest HaCaT cell migration rate of 5.886 ± 0.801 µg/h (*p* < 0.05) at 50 µg/mL after 96 h post-treatment (Figure 8). Similarly, a significant difference (*p* < 0.05) in the cell migration rate was also apparent between HaCaT cells treated with 75% *v*/*v* MeOH extract and allantoin (positive control), respectively, at the same treatment concentration (Figure 6). This further justifies the superior wound healing potency of 75% *v*/*v* MeOH extract relative to allantoin.

Cell migration is an essential step during the proliferative phase of wound healing, though it is an important mechanism involved in cancer metastasis. In a previous study by Kittiwattanokhun et al. [54], a CA leaf extract had been shown to significantly inhibit chondrosarcoma SW1353 cell migration in a scratch wound healing and transwell migration assay, in which the authors proposed its usefulness in chondrosarcoma metastasis treatment [64]. Based on the results from Figure 6, it was clearly noticeable that wound closures at 96 h were incomplete despite treatment at higher doses (100 and 200 µg/mL) of 75% *v*/*v* MeOH extract. Despite the insignificant effects on the cell viability (*p* > 0.05) of these two doses as demonstrated in Figure 5, Figure 8 showed significantly retarded HaCaT cell migration (*p* > 0.05) at doses higher than 50 µg/mL. Hence, the inhibition of HaCaT cell migration at higher doses in the study could be related to its migration inhibition property. In this current study, the delayed wound closure as observed in HaCaT cells (Figure 6) could be due to inhibition in cell migration (Figure 8) at higher doses. This shows that the wound closure and cell migration rate may not occur dose-dependently, contradicting the results reported by Kittiwattanokhun et al. [54]. This could be due to the types of cell lines used and their responses to the treatment.

Again, the wound-healing effect, particularly the cell migration rate of HaCaT cells treated with 75% *v*/*v* MeOH extract, was not dose dependent in this current study (Figure 8). This finding corroborates a previous study by Wedler et al. [65], who reported that higher concentrations of bamboo (*Phyllostachys edulis*) leaf extract could remarkedly inhibit the cell migration of mouse fibroblast 3T3 cells, while lower concentrations induced cell migration relative to the control. In the process of wound healing, the cell migration rate was in relation to the levels of vascular endothelial growth factor (VEGF), which assists in cell migration. VEGF secretion occurs in response to the release of inflammatory mediators like TNF-α. In their study, the authors proposed that due to the anti-inflammatory effect of the bamboo extract, higher doses of the extract could decrease TNF-α induced secretion of VEGF, hence inhibiting cell migration, while at lower doses of the extract, VEGF levels could be higher and thus promote cell migration [65]. Therefore, the less-favorable wound healing activity of 75% *v*/*v* MeOH extract at higher doses (100 and 200 µg/mL) could also be ascertained with its anti-inflammatory properties.

Referring to the HPLC analysis as shown in Table 1, the pronounced wound-healing properties of 75% *v*/*v* MeOH CA extract at 50 µg/mL could be a complex interplay between the flavonoids and anthraquinones characterized in this current study. Kaempferol, which has been widely studied for its wound-healing activity, was known to induce angiogenesis at low concentrations by increasing VEGF signaling, besides promoting VEGF-mediated HaCAT and RAW264.7 cell migration [66]. In murine models, kaempferol could reduce the formation of hypertrophic scarring induced by mechanical stress in mice and improve wound healing in both diabetic and nondiabetic rats [67,68]. Rhein, the major anthraquinone present in CA leaf extract was previously found to be able to increase HaCAT cell proliferation by binding with estrogen receptors [69]. Rhein has also been incorporated into hydrogels, which successfully accelerated the healing of *S. aureus*-infected wounds and diabetic wounds [70,71]. Aloe-emodin could also improve the rate of wound healing in murine burn wounds [72].

A prolonged inflammatory process in AD can result in serious skin lesions with delayed wound healing of the skin. In this current study, 75% *v*/*v* MeOH extract had been shown to exhibit outstanding wound-healing effects at a dose as low as 50 µg/mL. Along with the abundant presence of multiple constituents with proven wound-healing properties, *CA* leaves could make for a useful candidate for the treatment of wounds and skin lesions resulting from AD.

## 3. Materials and Methods

### 3.1. General Experimental Procedures

UV absorbances for antioxidant assays and the MTT assay were measured using a Tecan Infinite M Nano+ microplate reader (Zürich, Switzerland). HPLC analysis was carried out using an Agilent 1260 Infinity (Agilent Technologies, Mulgrave, Australia) instrument equipped with a quaternary pump, an autosampler, a thermostatic column compartment, and DAD. An inverted microscope TS100 (Nikon, Tokyo, Japan) equipped with NIS Elements software v5.01 was used to obtain microscopic images for scratch assay.

### 3.2. Chemicals

AR-grade MeOH and AR-grade DMSO were acquired from Fulltime Asia, Kuala Lumpur, Malaysia. HPLC-grade MeOH and di-potassium hydrogen phosphate, K_2_HPO_4_.3H_2_O, were acquired from Merck, Darmstadt, Germany. HPLC-grade quercetin (>95%), 2,2-diphenyl-1-picrylhydrazyl-hydrate (DPPH, 90%), C_18_H_12_N_5_O_6_, FBS (USA origin, sterile filtered, heat-inactivated), and trypsin-EDTA were acquired from Sigma Aldrich (St. Louis, MO, USA). HPLC-grade acetonitrile and HPLC-grade TFA were acquired from JT Baker (Deventer, The Netherlands). HPLC standards (astragalin, kaempferol, rhein, aloe-emodin) were acquired from Chengdu Alfa Biotechnology Co., Ltd., (Chengdu, China). Mueller–Hinton broth and Mueller–Hinton agar were obtained from Oxoid (Basingstoke, UK). Sodium bicarbonate, ampicillin sodium salt, MTT reagent (98.0%), and 1× PBS (Dulbecco A) were purchased from Chemsoln (Delhi, India). Iron (III) chloride-6-hydrate, FeCl_3_.6H_2_O (99.8%), was obtained from Fisher Scientific (Waltham, MA, UK). Potassium dihydrogen phosphate, KH_2_PO_4_, was obtained from Fisher Chemicals (Norristown, PA, USA). Folin–Ciocalteu’s phenol reagent and sodium carbonate anhydrous were obtained from Fluka (Buchs, Switzerland). Aluminium chloride, AlCl_3_.6H_2_O, was obtained from Bendosen (Ålen, Norway). Potassium acetate, CH_3_COOK, and trichloroacetic acid, CCl_3_COOH, were obtained from HmbG Chemicals (Hamburg, Germany). Potassium ferricyanide, K_3_Fe(CN)_6_ (99%), was obtained from Unilab (Mandaluyong, Philippines). DMEM (catalog number 12100046) was purchased from Thermofisher Scientific (Waltham, MA, USA). Penicillin–streptomycin was purchased from Sigma-Aldrich (St. Louis, MO, USA).

### 3.3. Plant Material and Extraction

*C. alata* leaves were collected from Yik Poh Ling Herbal Farm, Seremban, Negeri Sembilan, Malaysia. The identity of the leaves was established through the botanical characteristics as described [73]. The leaves were washed, blot-dried, and freeze-dried. The extract was obtained based on the procedure described by Cujic, et al. [74] and Ben Attia et al. [75], where the dried leaves were blended into powder using a blender, and then 50 g was dissolved with 1000 mL of aqueous MeOH solution at different concentrations (100%, 75%, and 50%). The mixture was macerated and left overnight at room temperature with continuous stirring. The extract was filtered, and the plant residue collected was re-extracted with the same solvent before filtration. The filtrates were dried using a rotary evaporator and a freeze dryer (Labconco 7382032 FreeZone 4.5L, Kansas City, MO, USA) to obtain leaf extracts. The extract was stored at −20 °C until further analysis was carried out.

### 3.4. HPLC–DAD Identification and Quantification of Major Constituents in Cassia alata Leaf Extracts

The extracts and standards were analyzed using a Supelco Analytical Ascentis^®^ C18 (25 cm × 4.6 mm, 5 µm) column, which was maintained at a temperature of 40 °C based on the validated method by de A Cavalcante et al. [76] with modifications. A 0.05% *v*/*v* trifluroroacetic acid (TFA) solution in water was used as solvent A and HPLC-grade acetonitrile as solvent B with a flow rate of 1 mL/min. The run process was as follows: initial 5% B, then 0–7 min: 5% B; 7–18 min: 5–30% B; 18–35 min: 30–60% B; 35–40 min, 60–95% B; 40–55 min, 95–5% B. The injection volume was 20 µL, and detection was carried out at 254 nm. The peak area in the chromatogram was used for the quantification of each standard in the extract. Different concentrations of the standard were run together with the extract to obtain the calibration curve for each standard, which was then used to quantify the constituent in each extract. The chromatograms and UV spectra obtained were analyzed using Openlabs Chemstation C.01.10.

### 3.5. In Vitro Antioxidant Evaluation of Cassia alata Leaf Extracts

#### 3.5.1. Total Phenolic Content (TPC)

The procedure was carried out based on the prior methodology [35,77]. A total of 150 µL of the diluted extract sample was transferred into a 24-well microplate in triplicate. A total of 750 µL of Folin–Ciocalteau phenol reagent (10% *v*/*v*) was added, followed by 600 µL of sodium carbonate (7.5% *w/v*), and then the plate was incubated in the dark for 30 min at room temperature. The UV absorbance was measured at 765 nm. TPC was expressed as mg GAE per 100 g of CA extract (mg GAE/100 g CA extract), and the standard curve used is y = 0.0081x (R^2^ = 0.9945), where y is absorbance and x is the concentration of gallic acid in mg according to Loh and Lim [35].

#### 3.5.2. Total Flavonoid Content (TFC)

The procedure was carried out based on a prior methodology [29,78,79,80] with some modifications. An amount of 0.5 mL of extract sample was prepared in triplicate in a clean dry test tube, then diluted with 1.5 mL of DMSO solvent. An amount of 0.1 mL of aluminum chloride (10% *w/v*) was added to each tube, followed by 0.1 mL of potassium acetate (1.0 M) and 2.8 mL of distilled water. The reaction mixture was incubated in the dark at room temperature for 30 min, and then 2 mL of the reaction mixture was transferred to a 24-well microplate. The absorbance was measured at 435 nm. The results were expressed in terms of mg RE per 100 g CA extract (mg RE/100 g CA extract) with the standard curve y = 0.019x (R^2^ = 0.9980) according to Chew, Goh, and Lim [29], where x represented the concentration of rutin in mg/L and y represented the absorbance measurement at 435 nm.

#### 3.5.3. 2,2-Diphenyl-1-picrylhydrazyl-hydrate (DPPH) Radical Scavenging Activity

The procedure was carried out based on a prior methodology [35,77]. An amount of 250 µL of the diluted sample in varying concentrations was transferred in triplicate onto a 24-well microplate, and then 1 mL of DPPH was added to each well. The reaction mixture was incubated in the dark for 30 min, and then the absorbance was measured at 517 nm. Scavenging activity was expressed as AEAC in mg AA per 100 g of CA extract (mg AA/100 g CA extract). AEAC was calculated as IC_50(ascorbate)_/IC_50(sample)_ × 10^5^, where the IC_50_ (concentration in mg/mL needed to reduce the initial absorbance of DPPH radicals by 50%) of ascorbic acid was 0.00408 mg/mL according to Loh and Lim [35].

#### 3.5.4. Ferric Reducing Antioxidant Power (FRAP)

The procedure was carried out based on a prior methodology [35,77]. An amount of 400 µL of extract sample in varying concentrations was transferred into test tubes. An amount of 1 mL of phosphate buffer (0.2 M, pH 6.6) was added to each tube, followed by 1 mL of potassium ferricyanide (1% *w/v*). After 20 min of incubation in a 50 °C water bath, 1 mL of trichloroacetic acid (TCA, 10% *w/v*) was added to terminate the reaction. Then, 1 mL aliquots of the resultant reaction mixture were transferred onto a 24-well microplate in triplicate, and then the mixture was diluted with 1 mL distilled water. An amount of 200 µL of ferric chloride (0.1% *w/v*) was added to each well, and then the mixture was incubated for 30 min in the dark. The absorbance was measured at 700 nm. FRAP was expressed as mg GAE per g of CA extract (mg GAE/g CA extract) based on the standard curve y = 21.498x (R^2^ = 0.9903) where y was absorbance and x was the concentration of gallic acid in mg/mL according to Loh and Lim [35].

#### 3.5.5. Ferrous Ion Chelating (FIC) Ability

The procedure was carried out based on a prior methodology [35,77]. An amount of 500 µL of extract samples of three varying dilutions was prepared in a 24-well microplate (100 µL extract + 400 μL DMSO solvent, 300 µL extract + 200 µL of DMSO solvent, 500 µL extract) in triplicate. An amount of 500 µL of freshly prepared iron (II) sulfate (0.1 mM) was added, followed by 500 µL of freshly prepared ferrozine (0.25 mM). The reaction mixture was incubated for 10 min in the dark, and then the absorbance was measured at 562 nm. The ability of the extract to chelate ferrous ions was calculated as FIC activity (%) = 1 − (A_sample_/A_control_) × 100, where A_sample_ and A_control_ are the absorbance of the sample and the control, respectively.

### 3.6. Evaluation of Antimicrobial Efficacy of Cassia alata Leaf Extracts Against Staphylococcus aureus ATCC 25923

*S. aureus* ATCC 25923 was obtained from the American Type Culture Collection (ATCC). Broth microdilution assay was used to determine the antimicrobial activity of the *C. alata* leaf crude extract against *S. aureus* ATCC 25923 according to the procedure by Rukayadi and Hwang [81] with slight modifications. The bacteria were incubated in Mueller–Hinton broth at 37 °C for 24 h, and the density of bacteria was standardized to approximately 1 × 10^7^ colony-forming units (cfu)/mL as standardized in the study carried out by Dusan [82]. MIC was determined by adding 100 μL of 20 mg/mL crude extract to the wells of a 96-well microplate, which was then diluted to the final concentrations of 10, 5, 2.5, 1.25, 0.625, 0.3125, 0.15625, and 0.078125 mg/mL. An amount of 100 μL of bacterial suspension was then added to the wells. A negative control of 0.25% DMSO and a positive control (ampicillin) were added to the wells. The microplate was incubated at 37 °C for 24 h. For MBC, 20 μL from each well that was visually clear was spotted on the Mueller–Hinton agar plate and incubated for 24 h at 37 °C. The tests were carried out in triplicate.

### 3.7. Cell Culture

HaCAT cell line was purchased from Elabscience, Houston, TX, USA. HaCaT cells were removed from liquid nitrogen and cultivated in complete DMEM containing 10% FBS and 1% penicillin–streptomycin. The cells were incubated at 5% CO_2_ and 37 °C until 70–80% confluency, upon which they were passaged into new cell flasks. The cell line was used for in vitro cell assays after two to three passages.

### 3.8. Evaluation of Cell Cytotoxicity

MTT assay was carried out based on a prior methodology by Thongrakard and Tencomnao [83] with modifications. HaCaT cells were seeded at a density of 5000 cells/well in a 96-well plate and incubated at 5% CO_2_ at 37 °C for 24 h. An amount of 100 μL extract of varying dilutions was then added to each well. Complete DMEM replaced the extract sample for the negative control, DMEM containing the highest concentration of DMSO from the extract samples replaced the samples as vehicle control, and 5% DMSO in DMEM replaced the extract sample for positive control. The mixture was then incubated for 24 h. MTT was first dissolved in 1x phosphate buffer saline (PBS) at 2 mg/mL concentration before dilution to 0.5 mg/mL using complete media. Media were discarded from the wells, 100 µL MTT solution was added to each well, and the plate was incubated in the dark for 3 h. The solution was then aspirated from the wells, and 100 μL of DMSO was added to each well to dissolve the crystals. The absorbance was read at 570 nm and 630 nm. The results were expressed as the percentage of cell viability (% CV), and the concentration of the extract, which resulted in 50% CV (IC50), was calculated.

### 3.9. In Vitro Wound-Healing Assay

The experiment was carried out based on prior methodology [84,85,86]. Briefly, HaCaT cells (5 × 10^4^) were seeded onto 24-well cell culture plates and allowed to grow to 90% confluence as a monolayer. Cells were starved with serum-free medium for 24 h, and then the monolayer was gently scratched with a sterile 200 µL pipette tip. After scratching, the culture medium was removed, and cellular debris was removed by washing with PBS solution. Serum-free medium with varying non-cytotoxic concentrations of the extract was added and incubated for 24 h at 37 °C in a 5% CO_2_ atmosphere. Cells without treatment and cells treated with allantoin (50 µg/mL) were used as the negative and positive controls, respectively. Images of the scratched cells were photographed at 0 and 24 h post-treatment. The wound areas and wound widths were measured using ImageJ v1.53t software, and results are expressed as means ± SD (*n* = 3). The percentage (%) of wound closure and migration rate was calculated according to the formulas as stated by Martinotti and Ranzato [84]. The experiments were conducted in triplicate. The results were analyzed using one-way ANOVA followed by post hoc Tukey’s HSD (Honestly Significant Difference) analysis.

### 3.10. Statistical Analysis

The results were analyzed using Microsoft Excel 2015 and IBM SPSS Statistics v20.0. All results for HPLC analysis, antioxidant activity, antimicrobial activity, MTT assay, and wound-healing activity were measured in triplicate (*n* = 3). Data were expressed as mean ± standard deviation (SD) wherever applicable.

## 4. Conclusions

In summary, this current study provides valuable insights into the potential benefits of CA in managing AD. This study successfully identified four important bioactive polyphenols (astragalin, kaempferol, rhein, aloe-emodin) in CA leaf extracts, with 75% *v*/*v* MeOH extract as the best extraction solvent selected based on the quantities of these polyphenols, supported by its antioxidant, antibacterial, and wound-healing efficacies that are potentially applicable in AD management. With relevance to AD, this study is the first to report the wound-healing properties of CA leaf extract in human skin cells. While promising, the research is limited by its exclusive focus on in vitro experiments, omitting in vivo and mechanistic investigations. Furthermore, this study also does not involve human subjects, making it challenging to directly apply these findings to AD management in clinical settings. Hence, future research should expand into in vivo and clinical studies while exploring the mechanism of action of CA leaf extracts to establish a stronger scientific foundation for using CA leaves in AD management in real-world scenarios.

## Figures and Tables

**Table 1:** Concentration of important bioactive constituents detected in CA extracts.

Chemical Constituent	Concentration (µg/mg)		
	100% *v*/*v* MeOH CA Extract	75% *v*/*v* MeOH CA Extract	50% *v*/*v* MeOH CA Extract
Astragalin	54.0 ± 1.92 ^a^	66.0 ± 0.357 ^b^	60.7 ± 0.315 ^c^
Kaempferol	16.4 ± 0.100 ^a^	25.2 ± 0.100 ^b^	22.4 ± 0.050 ^c^
Aloe-emodin	77.2 ± 0.342 ^a^	121 ± 0.388 ^b^	101 ± 0.246 ^c^
Rhein	157 ± 0.217 ^a^	174 ± 0.407 ^b^	163 ± 0.235 ^c^

Results are presented as mean ± standard deviation (SD) (*n* = 3). For each row, values followed by the same letter are not statistically different (*p* < 0.05) as measured using one-way analysis of variance (ANOVA) followed by post hoc Tukey’s Honest Significant Difference (HSD) analysis.

**Table 2:** Antioxidant properties of CA extracts prepared using 100% *v*/*v* MeOH, 75% *v*/*v* MeOH, and 50% *v*/*v* MeOH.

Samples	TPC (mg GAE/100 g CA Extract)	TFC (mg RE/100 g CA Extract)	Antioxidant Activity		
			IC_50_ (mg/mL)	AEAC (mg AA/100 g CA Extract)	FRAP (mg GAE/g CA Extract)
100% *v*/*v* MeOH CA Extract	7440 ± 15.7 ^a^	1680 ± 34.8 ^a^	0.521 ± 0.025 ^a^	784 ± 36.7 ^a^	23.9 ± 0.60 ^a^
75% *v*/*v* MeOH CA Extract	8190 ± 83.7 ^b^	1710 ± 16.2 ^a^	0.446 ± 0.009 ^b^	915 ± 19.2 ^b^	22.1 ± 0.47 ^b^
50% *v*/*v* MeOH CA Extract	7160 ± 47.2 ^c^	922 ± 27.7 ^b^	0.555 ± 0.024 ^a^	736 ± 31.2 ^a^	19.8 ± 0.29 ^c^

Results are presented as mean ± SD (*n* = 3). Values followed by the same letter are not statistically different (*p* < 0.05) as measured using one-way ANOVA followed by post hoc Tukey’s HSD analysis. TPC, total phenolic content; TFC, total flavonoid content; IC_50_, inhibitory concentration of DPPH radicals at 50%; AEAC, ascorbic acid equivalent antioxidant capacity; FRAP, ferric reducing antioxidant power; GAE, gallic acid equivalent; RE, rutin; AA, ascorbic acid.

**Table 3:** Antimicrobial efficacy of CA extracts prepared using 100% *v*/*v* MeOH, 75% *v*/*v* MeOH, and 50% *v*/*v* MeOH against *S. aureus* ATCC 25923.

Sample	MIC (mg/mL)	MBC (mg/mL)
100% *v*/*v* MeOH CA Extract	1.25	5.00
75% *v*/*v* MeOH CA Extract	0.625	1.25
50% *v*/*v* MeOH CA Extract	1.25	>5.00
Ampicillin (Positive Control)	7.81 × 10^−5^	7.81 × 10^−5^

## Data Availability

Data are contained within the article.
